# Participant Service Ability Aware Data Collecting Mechanism for Mobile Crowd Sensing

**DOI:** 10.3390/s18124219

**Published:** 2018-12-01

**Authors:** Jing Yang, Jialiang Xu

**Affiliations:** 1 School of Communication and Information Engineering, Chongqing University of Posts and Telecommunications, Chongqing 400065, China; yangjingb@cqupt.edu.cn; 2 Key Laboratory of Optical Communication and Networks, Chongqing 400065, China; 3 Key Laboratory of Ubiquitous Sensing and Networking, Chongqing 400065, China

**Keywords:** Mobile Crowd Sensing, data collecting, Stackelberg game, service ability

## Abstract

To collect data efficiently and reliably in Mobile Crowd Sensing (MCS), a Participant Service Ability Aware (PSAA) data collecting mechanism is proposed. First, participants select the best sensing task according to the task complexity and desired reward in the multitasking scenario. Second, the Stackelberg Game model is established based on the mutual choice of participants and platform to maximize their utilities to evaluate the service ability of participants. Finally, participants transmit data to platform directly or indirectly through the best relay and the sensing data from the participants with better service ability is selected to complete sensing tasks accurately and efficiently with the minimum overall reward expense. The numerical results show that the proposed data collection mechanism can maximize the utility of participants and platform, efficiently accomplish sensing tasks and significantly reduce the overall reward expense.

## 1. Introduction

In recent years, the rapid proliferation of mobile devices such as smartphones and tablets with powerful sensing ability enables MCS to become a mobile application hotspot for the collaborative work between participants and platform [1,2]. In MCS, participants exploit the sensors embedded in their carried mobile devices (e.g., light sensor and magnetic force sensor) to collect and share data including the ambient brightness and magnetic field [3,4,5]. Then, the platform gathers sensing data from participants and provides real time and accurate services based on the sensing results [6]. Because MCS collects large-scale sensing data efficiently and flexibly, it can be applied to various projects. OrganiCity [7] is an example project with a very specific purpose to engage people in the development of future smart cities and provides an Experimentation as a Service (EaaS) platform to provide data streams from diverse sources inside a smart city to consumers, while recruiting participants to collect data for updating diverse sources. SmartSantander [8] is based on the largest Future Internet (FI) infrastructure and creates an experimental test facility for the research of Internet of Things (IoT) architecture, services and applications. Participants can integrate smartphones into the SmartSantander infrastructure to not only extend the infrastructure capabilities but also expand the sensing coverage and ubiquity. Besides, SmartSantander can use these smartphones for event detection. For example, SmartSantander can study mobility patterns in city streets and simultaneously monitor environmental parameters. YouSense [9] is a model-based platform created to manage participatory sensing activities and actively notify participants with questions when they are in the optimal location to collect sensing data. While avoiding bothering participants with unnecessary requests, YouSense motivates more participants to actively participate in sensing activities to increase the quantity and quality of sensing data.

In MCS, the platform is responsible for publishing sensing tasks and providing certain rewards to motivate participants to actively perform sensing tasks. Participants are responsible for exploiting sensors embedded in mobile devices to collect and upload data to the platform [10]. Compared with the platform, participants are service providers, so the ability of participants to complete the sensing task accurately and efficiently can be defined as service ability, and the high service ability indicates that they can complete sensing tasks more efficiently. Therefore, the service ability is an important factor for participants to complete sensing tasks accurately and efficiently. However, the service ability evaluation and participant selection become challenging problems.

The existing MCS data collection research has two application modes according to service objects: participant-centric and platform-centric modes [11,12]. In the participant-centric mode, the relevant sensing data are collected by mobile devices to record participant activities. In contrast, the platform-centric mode focuses on collecting environmental data and publishing sensing results online to meet the public demands after analyzing and processing the sensing data. Although the above modes can collect some sensing data, they only consider the number of participants in sensing tasks or the total volume of sensing data collected by the platform. The larger number of participants and greater total volume of sensing data signify the higher probability to complete sensing tasks, but these two modes ignore the participant service ability. However, in the multitasking scenario, there are differences among participants in several factors, such as the geographical location, interest and residual energy, resulting in the differences in task duration and overall reward expense [13,14,15]. Therefore, how to exploit participants with better service ability in sensing tasks, how to select sensing tasks according to the service ability of participants and how to complete sensing tasks accurately and in real time with the minimum overall reward expense become key technical challenges.

In view of the above problems, a PSAA data collection mechanism is proposed. First, participants measure the task complexity according to their willingness, energy consumption rate and task duration, and then select the best sensing task based on the desired reward. Meanwhile, to maximize the utility of platform and participants, the platform evaluates the service ability of participants according to the timeliness and desired reward of participants and complexity of sensing tasks to selectively collect sensing data. To complete sensing tasks efficiently, participants determine the transmission mode of sensing data according to their residual energy and task duration, and then transmit data to the platform directly or indirectly through the best relay, so that sensing tasks are accomplished accurately and in real time with the minimum overall reward expense.

The main contributions of this paper are as follows:

(1) The complexity of sensing task is evaluated by the participant willingness, energy consumption rate and task duration to effectively improve the energy utilization of mobile devices performing sensing tasks. Consequently, participants select the best task according to the task complexity and desired reward.
(2) Aiming at the mutual choice between participants and platform, the Stackelberg game model is established to maximize the utility of participants and platform. Furthermore, the participant service ability is evaluated according to the participant’s utility, timeliness, desired reward and task complexity, and employed by the platform to selectively receive sensing data.
(3) To collect sensing data in time and provide reliable service, participants determine whether the sensing data are transmitted directly or indirectly to the platform based on the residual energy, energy consumption rate and task duration. If participants forward sensing data indirectly to the platform, the best relay is selected based on the participant service ability, sensing task similarity and intimacy degree between participants.

The rest of the paper is organized as follows. The related work are introduced in Section 2. Section 3 describes the system model. The participant service ability is evaluated in Section 4. Section 5 designs a PSAA data collection mechanism. The numerical results are given in detail in Section 6. Finally, Section 7 concludes this paper.

## 2. Related Work

We now introduce related work on MCS data collecting mechanisms.

Many research efforts have been dedicated to MCS data collecting mechanisms. An incentive-aware time-sensitive data collection mechanism is designed in [16]. The proposed mechanism selects relay user by applying the Nash bargain solution, and participants forward sensing data through relay users to ensure the timely data collection. Although the above mechanism can motivate participants to collect sensing data actively, it ignores the overall reward expense of the platform. In addition, Shah-Mansouri et al. [17] proposed a Profit Maximizing Truthful (ProMoT) auction mechanism. In ProMoT, the platform releases sensing tasks to participants, and then participants submit task bids to the platform. Then, the platform selects a subset of participants based on their bids and provides proper payments for them. Although the ProMoT auction mechanism can maximize the platform utility, reduce the overall reward expense and motivate participants to truthfully participate in the auction, it ignores the data quality of participants and cannot guarantee the accuracy and reliability of sensing data. Sun et al. [18] designed an incentive scheme based on heterogeneous trust values for joint social states and real-time throughput. Participants are selected according to state attributes. Nevertheless, the proposed mechanisms do not take the reliability of sensing data into account in [16,17,18]. Because participants are affected by their attributes, social behaviors and other factors in MCS, when participants collect low quality sensing data, the sensing tasks cannot be completed accurately and reliably.

To reliably collect sensing data in MCS, Gao et al. [19] designed a data quality prediction mechanism with Poisson distribution based on the assumption that participants transmitted sensing data one by one. The proposed mechanism ensures the high quality of data collection and completed sensing tasks accurately. Dai et al. [20] proposed the Integrated Incentive Mechanism (IIM) to motivate participants to provide high quality sensing data, where the platform updates the reputation of participants based on their corresponding behaviors. Wen et al. [21] designed a quality-driven auction-based incentive mechanism to evaluate the reliability of sensing data according to a probabilistic model and verified the rationality of the proposed mechanism in the indoor positioning application scenario. Krontiris et al. [22] proposed a multi-attribute auction mechanism based on the traditional auction mechanism, which comprehensively considered factors such as the rewards of participants, durations of sensing tasks and total amount of collected sensing data to effectively improve the reliability of sensing data. Although the above mechanisms can ensure the reliability of sensing data, they ignore the timeliness of sensing tasks and cannot meet the flexibility and real time requirements of MCS.

In addition, only three factors are considered by the above-mentioned methods, namely the timeliness of sensing tasks, reliability of sensing data and reward expense of platform, but the complexity of sensing tasks should also be considered. In MCS, if the complexity of sensing tasks exceeds the sensing ability of participants, they cannot complete the sensing tasks efficiently and accurately. Given this kind of situation, a data collecting mechanism is designed in this paper to balance the complexity, timeliness of sensing tasks and platform utility to ensure the timely accomplished sensing tasks with the minimum overall reward expense.

## 3. System Model

**Figure 1:**
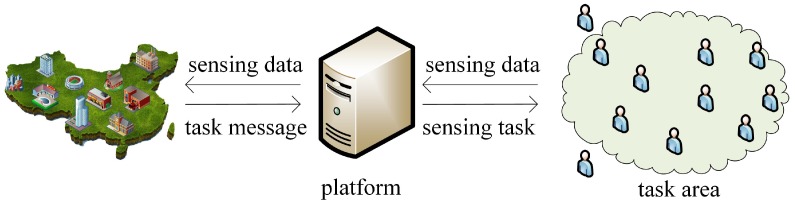
Data collection process.

The data collection process is shown in Figure 1. When users need to collect some GPS data, they send the task request to platform, and then the platform analyzes and sends sensing tasks to participants. In the multitasking scenario, when participants receive the information of sensing tasks in the task area, they select the best task by evaluating the complexity of sensing tasks and then collect sensing data correspondingly. At the same time, through the comprehensive analysis of the utility of participants and platform, the timeliness and other factors are combined to evaluate the participant service ability, and then the platform chooses participants with high sensing data quality and low rewards. In the participant selecting process, the best sensing task and participants with better service ability are chosen, where the utility of participants and platform can be maximized simultaneously by the equilibrium solution. After sensing data are transmitted directly or indirectly to the platform, the platform decides whether to receive the sensing data according to the service ability, and therefore can complete sensing tasks reliably and in time with the minimum overall reward expense.

In the above process, the GPS data must differ among distinct locations. To tackle the spatial inconsistencies, the task message is divided into many sensing tasks. We assume that the set of sensing tasks is denoted by $F=\{f_{1},\;\;f_{2},...,\;\;\;\;f_{n}\}$, each sensing task has its specified area and time [23]. Considering that some participants may require some extra effort for performing sensing tasks, i.e., participants need to position themselves at the specified area. However, this paper focuses on how participants choose sensing tasks and how the platform selectively receives sensing data based on the service ability of participants when they are in the task area. Therefore, $P=\{p_{1},\;\;\;\;p_{2},...,\;\;\;p_{m}\;\}$ is denoted by the set of participants within the task area, the participant $p_{i}$ can measure the complexity of sensing tasks and select the best sensing task. At the same time, when $p_{i}$ performs the sensing task, the ongoing work or battery life of $p_{i}$ may be affected. To avoid the above situation, $p_{i}$ is free to choose the time to perform the sensing task, but he must complete the sensing tasks within the task deadline. In addition, we note that, if the sensing data of $p_{i}$ are constantly rejected by the platform, his willingness to perform the sensing task will be reduced. To actively motivate $p_{i}$ participations in MCS, if the service ability of $p_{i}$ is the same as others, the platform will receive his sensing data preferentially.

## 4. Evaluation of Service Ability

First, participants measure the complexity of a sensing task based on the willingness of participants, energy consumption rate of mobile devices and duration of sensing tasks, and then the best task can be selected according to the desired reward. Second, the Stackelberg game model is established to analyze the utility of participants and platform to maximize the reward of participants and the overall reward expense utilization of the platform in the equilibrium solution. Finally, by evaluating the service ability of participants, the platform selects participants with better service ability and participants choose the best task to complete accurately and reliably.

### 4.1. The Best Task Decision

Participants only execute one sensing task under the multitasking scenario. Because participants choose sensing tasks according to their willingness, if the willingness of participants is low, the probability of refusing a sensing task is high, resulting in the high complexity of the sensing task. In addition, since the collection of sensing data mainly depends on the sensors embedded in mobile devices, different types of sensing tasks should be processed. When collecting sensing data, mobile devices carried by participants suffer a certain energy loss. If a participant consumes more energy for a given sensing task, its complexity is higher. When participants perform a sensing task with long duration, they not only consume more energy in the processes of collecting, storing and transmitting sensing data, but also affect the real time performance of tasks, and therefore the task complexity is high. Based on the above analysis, the complexity of sensing tasks should be defined by the participant willingness, energy consumption rate and task duration.

We assume the sensing task ultimately chosen by participant $p_{i}$ is $f_{j}$. If the time when the platform allows participants to start $f_{j}$ is $t_{f_{j}}^{s}$ and the time when sensing data collection ends is $t_{f_{j}}^{e}$ , $t_{f_{j}}^{e}-t_{f_{j}}^{s}$ is the task duration. Besides, we suppose the time when $p_{i}$ receives the information of $f_{j}$ is $t_{ij}^{a}$ and the time when $p_{i}$ begins to collect sensing data is $t_{ij}^{m}$. Obviously, the value range of $t_{ij}^{m}$ is $\left[t_{ij}^{a},\;\;\;\;t_{f_{j}}^{e}\right]$, participating decision time $B_{ij}$ of $p_{i}$ to $f_{j}$ is $t_{ij}^{m}-t_{ij}^{a}$. If participating decision time $B_{ij}$ is short, $p_{i}$ will participate in sensing task $f_{j}$ actively, and the willingness of $p_{i}$ is high. In addition, We define that the total energy of participant $p_{i}$ is $E_{i}^{a}$, his residual energy is $E_{i}^{r}$. Since $p_{i}$ may affect the use of mobile devices, the high the value of $E_{i}^{r}$ signifies the active participation of $p_{i}$ in $f_{j}$, namely the high willingness of $p_{i}$. In addition, if the decision time $B_{ij}$ is equal to 0, it can be judged that the willingness of $p_{i}$ is equal to 1, which means that the sensing task is performed immediately when $p_{i}$ receives the information of $f_{j}$. Based on the above analysis, the willingness $W_{ij}$ of $p_{i}$ can be measured by decision time $B_{ij}$ and residual energy $E_{i}^{r}$, as calculated by Equation (1).

(1) Wij=EirEia·∑i=1vBij∑i=1vBijvvBij,Bij≠01,Bij=0

where *v* is the total number of participants selecting task $f_{j}$ and ∑i=1vBij∑i=1vBijvv is the average decision time for $f_{j}$.

The willingness of $p_{i}$ can be effectively measured by Equation (1). Furthermore, the energy consumption per unit time of $p_{i}$ is assumed to be $\gamma_{ij}$ in sensing task $f_{j}$, so that complexity $D_{ij}$ of sensing task $f_{j}$ measured by $p_{i}$ is obtained by Equation (2).

(2) $$D_{ij}=1-W_{ij}+\frac{\gamma_{ij}\cdot \left(t_{f_{j}}^{e}-t_{ij}^{m}\right)}{E_{i}^{r}}$$

To maximize the actual rewards with the minimum energy consumption, participants choose the best task. Assuming the desired reward of $p_{i}$ is $c_{ij}$ according to $D_{ij}$ , $p_{i}$ selects the best task based on Equation (3):

(3) fj=arg∀fj∈Fmax(xij·cijcijDijDij)xij·(1−xij)=0∑∀fj∈Fxij=1

where cijcijDijDij is the degree that $p_{i}$ participates in $f_{j}$ . When desired reward $c_{ij}$ of $p_{i}$ is high, the complexity $D_{ij}$ of $f_{j}$ is low. To maximize the reward with the minimum energy consumption, a large cijcijDijDij signifies a high probability of $p_{i}$ participating in $f_{j}$. Moreover, $x_{ij}$ is a binary Boolean variable and $x_{ij}$ is set to 1 when $p_{i}$ selects task $f_{j}$. Otherwise, $x_{ij}$ is set to 0. $\sum_{\forall f_{j}\in F}x_{ij}=1$ ensures the total number of sensing tasks performed by $p_{i}$ at the same time is at most 1.

### 4.2. Utility Analysis

When participants perform the best task, there is an energy loss in their mobile devices during the sensing data collecting process. Therefore, when participants perform sensing tasks successfully, the platform needs to pay some rewards to compensate for the energy loss to motivate participants to perform sensing tasks actively. However, the overall reward expense of each sensing task is limited, so participants with the high sensing data quality and low actual rewards must be selected to minimize the overall reward expense of the platform.

#### 4.2.1. Data Quality

Sensing data quality is determined based on two factors: completeness and accuracy. Because the more complete are the sensing data, the clearer is the information transmitted to the platform by participants, data completeness is conducive to the accurate completion of sensing tasks. In addition, the sensing data accuracy can reflect the ground truth, which is also beneficial to the accurate completion of the sensing task.

We suppose that the total volume of sensing data acquired by $p_{i}$ in the sensing task $f_{j}$ is $l_{ij}$. Theoretically, the total volume of data obtained by $p_{i}$ is $l_{ij}^{total}$. Obviously, the completeness of sensing data is high when $l_{ij}$ approaches $l_{ij}^{total}$ and max(lijlijlijtotallijtotal) signifies the maximum approximation among all participants selecting task $f_{j}$, so the completeness $h_{ij1}$ of sensing data can be calculated by:

(4) hij1=lijlijlijtotallijtotalmax(lijlijlijtotallijtotal)

For the accuracy of sensing data, we assume that, when the platform ends collecting sensing data, the sensing dataset collected by the participants selecting task $f_{j}$ is $X_{j}=\left\{x_{1j},x_{2j},...,x_{vj}\right\}$ and the platform will find a sensing data $y_{j}$ with the highest similarity from $X_{j}$. When $x_{ij}$ is close to $y_{j}$, the similarity between $x_{ij}$ and $y_{j}$ is high, indicating that the values of $x_{ij}$ and $y_{j}$ are close and the accuracy of $x_{ij}$ is high. Therefore, the accuracy $h_{ij2}$ of $x_{ij}$ is measured according to the similarity between $x_{ij}$ and $y_{j}$, as shown in Equation (5).

(5) $$h_{ij2}=\frac{\frac{\theta }{{\left(y_{j}-x_{ij}\right)}^{2}}}{\sum_{z=1}^{m}\frac{\theta }{{\left(y_{j}-x_{zj}\right)}^{2}}}$$

where the value of ${\left(y_{j}-x_{ij}\right)}^{2}$ signifies the similarity between $x_{ij}$ and $y_{j}$, a small value indicates a high similarity, θ is the sum of similarity deviations, and $\theta =\sum_{i=1}^{v}{\left(y_{j}-x_{ij}\right)}^{2}$ .

In the calculation of $y_{j}$, the accuracy of each sensing data is initialized to 1/v to measure the weight of $y_{j}$. Thus, the most similar sensing data $y_{j}$ can be obtained by Equation (6).

(6) $$y_{j}=\arg\min\sum_{i=1}^{v}\left(h_{ij2}\cdot {\left(y_{j}-x_{ij}\right)}^{2}\right)$$

where $h_{ij2}$ is obtained by the iterative calculation. First, $h_{ij2}$ is initialized to 1/v, and $y_{j}$ is calculated according to Equation (6). Then, $h_{ij2}$ is updated by Equation (5). Finally, when the iteration converges, the update stops and the latest updated value is the accuracy of sensing data.

Through the above analysis, data quality $q_{ij}$ of $p_{i}$ can be quantified from completeness $h_{ij1}$ and accuracy $h_{ij2}$. Since $h_{ij1}$ and $h_{ij2}$ have different effects on $q_{ij}$, data quality of $p_{i}$ can be quantified by $q_{ij}=\alpha_{j1}h_{ij1}+\alpha_{j2}h_{ij2}$, where $\alpha_{j1}+\alpha_{j2}=1$.

To avoid subjective factors leading to inaccurate results of $\alpha_{j1}$ and $\alpha_{j2}$, we use the entropy weight method to determine their values [24]. The data dimension of $h_{ij1}$ and $h_{ij2}$ may introduce errors, so $h_{ij1}$ and $h_{ij2}$ are standardized, as shown in Equation (7).

(7) $$\overset{\wedge }{h_{ijL}}=\frac{h_{ijL}-\mu_{jL}}{o_{jL}},L=1,2$$

where $\mu_{jL}$ and $o_{jL}$ represent the mean and standard deviation of $h_{ijL}$, respectively.

We can obtain the information entropy $H_{jL}$ of $h_{ijL}$ according to Equations (7) and (8).

(8) $$H_{jL}=-\frac{1}{\ln v}\cdot \sum_{i=1}^{v}\vartheta_{ijL}\ln\vartheta_{ijL}$$

where $\vartheta_{ijL}$ is the proportion of $\overset{\wedge }{h_{ijL}}$ and ϑijL=h∧ijLh∧ijL∑i=1vh∧ijL∑i=1vh∧ijL. Finally, the normalized weight $\alpha_{jL}$ of $h_{ijL}$ can be calculated by:

(9) αjL=αjL′αjL′∑L=12αjL′∑L=12αjL′

where $\alpha_{jL}^{{}^{\prime }}$ is the entropy weight of $H_{jL}$ and αjL′=(1−HjL)(1−HjL)∑L=12(1−HjL)∑L=12(1−HjL).

#### 4.2.2. Utility Analysis

The platform can analyze the utility of participants based on the data quality and actual rewards, and then select participants with high utilities to maximize the platform utility. We assume that the overall reward expense of platform for sensing task $f_{j}$ is $R_{j}$, and the energy cost per data quality is $o_{j}$ in sensing task $f_{j}$. If the data quality of $p_{i}$ is $q_{ij}$, the utility $u_{j}^{p_{i}}\left(q_{ij},\;\;\;m_{p}\right)$ of $p_{i}$ to $f_{j}$ can be calculated by:

(10) $$u_{j}^{p_{i}}\left(q_{ij},\;\;\;m_{p}\right)=m_{p}\cdot \left(q_{ij}/\sum_{p_{i}\in P^{j}}q_{ij}\right)R_{j}-o_{j}\cdot q_{ij}$$

where $m_{p}$ is the parameter determining the sensing data value, $P^{j}$ is the set of participants performing sensing task $f_{j}$, and $m_{p}\cdot \left(q_{ij}/\sum_{p_{i}\in P^{j}}q_{ij}\right)R_{j}$ indicates the actual reward of $p_{i}$.

In situations of few participants in a sensing task, the sensing data quality of participants is crucial for the platform to accomplish the sensing task accurately and reliably. Because of the limited participant range, the platform utility grows with the increasing sensing data quality. With the increasing number of participants, the platform aims to achieve the minimum overall reward expense. By not only considering the sensing data quality of participants but also analyzing the utility of participants, the platform prefers to select participants with high utility. Therefore, when the number of participants is large, the sensing data quality is no longer the only factor affecting the accurate and reliable completion of sensing tasks, and the platform utility grows slowly with the increasing sensing data quality. Based on characteristics of platform utility and its change with sensing data quality, platform utility $u_{j}^{s}\left(q_{ij},\;\;\;m_{p}\right)$ for sensing task $f_{j}$ can be calculated by Equation (11).

(11) $$u_{j}^{s}\left(q_{ij},\;\;\;m_{p}\right)=m_{s}\cdot \log_{2}\left(1+\sum_{p_{i}\in P^{j}}q_{ij}\right)-m_{p}\cdot R_{j}$$

where $m_{s}$ is the parameter determining the value of the received sensing data and $\log_{2}\left(1+\sum_{p_{i}\in P^{j}}q_{ij}\right)$ reflects the overall trend of platform utility with the increase of data quality.

Since participants expect to maximize rewards with the minimal energy consumption, it is necessary to maximize the utility of participants by solving the optimization problem in Equation (12).

(12) $$\begin{array}{c} \max_{q_{ij}}\;\;\;\;m_{p}\cdot \frac{q_{ij}}{\sum_{p_{i}\in P^{j}}q_{ij}}R_{j}-o_{j}\cdot q_{ij}\\ s.t.\;\;\;\;q_{ij}\geq 0 \end{array}$$

Correspondingly, the platform selects participants with high sensing data quality and low actual rewards to minimize the overall reward expense. Therefore, it is necessary to maximize the platform utility by addressing the optimization problem in Equation (13).

(13) $$\begin{array}{c} \max_{m_{p}}\;\;\;\;\;m_{s}\cdot \log_{2}\left(1+\sum_{p_{i}\in P^{j}}q_{ij}\right)-m_{p}\cdot R_{j}\\ s.t.\;\;\;\;m_{p}\geq 0 \end{array}$$

Obviously, Equations (12) and (13) constitute a Stackelberg game [25,26]. Since Stackelberg equilibrium belongs to subgame Nash equilibrium, the unilateral decision changes of participants or platform cannot further improve their utilities. Consequently, the equilibrium must be obtained to find a stable utility equilibrium between participants and platform, so that the utilities of both are maximized. According to the definition of equilibrium solution [27], for any $q_{ij}\geq 0$ and $m_{p}\geq 0$, if $\left(q_{ij}^{*},m_{p}^{*}\right)$ satisfies the conditions in Equation (14), $\left(q_{ij}^{*},m_{p}^{*}\right)$ is the equilibrium solution of the Stackelberg game.

(14) $$\left\{\begin{array}{c} u_{j}^{p_{i}}\left(q_{ij}^{*},\;\;\;\;\;m_{p}^{*}\right)\geq u_{j}^{p_{i}}\left(q_{ij},\;\;\;\;\;m_{p}^{*}\right)\\ u_{j}^{s}\left(q_{ij}^{*},\;\;\;\;\;m_{p}^{*}\right)\geq u_{j}^{s}\left(q_{ij}^{*},\;\;\;\;\;m_{p}\right) \end{array}\right.$$

According to Equations (10) and (13) and the number of participants in the sensing task, Equation (15) can be obtained.

(15) $$q_{ij}^{*}=m_{p}\cdot \frac{\left|P^{j}\right|-1}{{\left|P^{j}\right|}^{2}\cdot o_{j}}R_{j}$$

where $q_{ij}^{*}$ is an expression of parameter $m_{p}$ and $\left|P^{j}\right|$ represents the total number of participants in the set of $P^{j}$. According to Equations (11) and (15), the partial derivatives of platform utility $u_{j}^{s}$ can be exploited to find the maximum value, as shown in Equation (16).

(16) $$\left\{\begin{array}{c} \frac{\partial u_{j}^{s}}{\partial m_{p}}=\frac{m_{s}}{\ln2}\cdot \frac{\left(\left|P^{j}\right|-1\right)R_{j}}{\left|P^{j}\right|\cdot o_{j}+m_{p}\cdot \left(\left|P^{j}\right|-1\right)R_{j}}-R_{j}\\ \frac{\partial^{2}u_{j}^{s}}{\partial m_{p}^{2}}=-\frac{m_{s}}{\ln2}\cdot \frac{{\left(\left|P^{j}\right|-1\right)}^{2}R_{j}^{2}}{{\left[\left|P^{j}\right|\cdot o_{j}+m_{p}\cdot \left(\left|P^{j}\right|-1\right)R_{j}\right]}^{2}} \end{array}\right.$$

When $m_{p}$ is equal to zero, obviously $u_{j}^{s}\left(q_{ij},\;\;\;m_{p}\right)$ is also zero, so platform utility $u_{j}^{s}\left(q_{ij},\;\;\;m_{p}\right)$ is equal to zero according to Equation (11). Because $u_{j}^{s}\left(q_{ij},\;\;\;m_{p}\right)$ is increasing slowly, when ${\left.u_{j}^{s}\left(q_{ij},\;\;\;m_{p}\right)\right|}_{m_{p}=0}=0$, ${\left.\frac{\partial u_{j}^{s}}{\partial m_{p}}\right|}_{m_{p}=0}>0$ can be obtained and we can determine the value range of $m_{s}$, as shown in Equation (17).

(17) $$m_{s}>\ln2\cdot \frac{\left|P^{j}\right|\cdot o_{j}}{\left|P^{j}\right|-1}$$

Since Equation (16) shows that $\frac{\partial^{2}u_{j}^{s}}{\partial m_{p}^{2}}\leq 0$ always holds, parameter $m_{p}$ satisfying $\frac{\partial u_{j}^{s}}{\partial m_{p}}=0$ can maximize $u_{j}^{s}\left(q_{ij},\;\;\;m_{p}\right)$, where $m_{p}^{*}$ can be calculated by:

(18) $$m_{p}^{*}=\frac{m_{s}}{\ln2\cdot R_{j}}-\frac{\left|P^{j}\right|\cdot o_{j}}{\left(\left|P^{j}\right|-1\right)R_{j}}$$

Thus, the equilibrium solution can maximize the utility of participants and platform, as shown in Equation (19).

(19) $$\left\{\begin{array}{c} u_{j}^{p_{i}}\left(q_{ij}^{*},\;\;\;\;\;m_{p}^{*}\right)=\frac{m_{p}^{*}\cdot R_{j}}{{\left|P^{j}\right|}^{2}}\\ u_{j}^{s}\left(q_{ij}^{*},\;\;\;\;\;m_{p}^{*}\right)=m_{s}\cdot \log_{2}\left(\frac{\left|P^{j}\right|-1}{\left|P^{j}\right|\cdot o_{j}}\cdot \frac{m_{s}}{\ln2}\right)-\frac{m_{s}}{\ln2}+\frac{\left|P^{j}\right|\cdot o_{j}}{\left|P^{j}\right|-1} \end{array}\right.$$

According to Equation (19), as the total number of participants $\left|P^{j}\right|$ increases, the utility of participants decreases sharply, because, when $\left|P^{j}\right|$ is large enough, the platform will selectively receive sensing data to provide accurate and reliable services. Therefore, to incite the platform to receive their sensing data, participants will inevitably improve sensing data quality or reduce actual rewards. However, when participants change their strategies unilaterally, they cannot improve their own utilities in the Stackelberg game, so that the utility of participants decreases as $\left|P^{j}\right|$ increases. Moreover, as shown in Equation (19), parameter $m_{s}$ in the platform utility $u_{j}^{s}\left(q_{ij}^{*},m_{p}^{*}\right)$ are not all positive, because $m_{s}$ determines the value of the received sensing data. As the quality of sensing data is greatly improved, the platform utility is not sharply increased, so $m_{s}$ are not all positive in $u_{j}^{s}\left(q_{ij}^{*},m_{p}^{*}\right)$. Based on the above analysis, the game result determined by the equilibrium solution is accurate, which can maximize the utility of participants and platform.

### 4.3. Evaluation of Service Ability

The utilities of participants and platform are maximized to select the best task in the multitasking scenario. However, due to the significant features of a large number of participants in MCS, the only goal of participants is to maximize their actual rewards in the sensing data collecting process and they ignore the final rewards of other participants performing the same task. At the same time, the only goal of the platform is to provide accurate and reliable services with the minimum overall reward expense. Therefore, the maximum utility of a single participant or the platform cannot unilaterally achieve the optimal overall utility. In addition, because participants have random mobility, they may be interfered by factors such as the geographical environment in the sensing data collecting process, causing the sensing data to be inconsistent with task requirements or to be tampered with by malicious participants. The above situation seriously affects the sensing results of the platform, and therefore not only participants should choose the best task, but also the platform should screen all participants performing the same sensing task to provide accurate and reliable services.

Participants have the great flexibility in choosing a sensing task at any time, the willingness of participants varies, and the energy consumption of sensing tasks is also different, indicating participants independently measure the complexity of sensing tasks. Moreover, to maximize the actual rewards of participants, minimize the overall reward expense of platform, and maximize the utilities of participants and platform as a whole, the platform needs to analyze the service ability of participants to select the best participants. The service ability is crucial for the platform to measure whether to receive the sensing data of participants; a stronger service ability signifies a higher probability of sensing data being received by the platform.

The platform limits the duration of sensing tasks due to the real time requirements of services. Obviously, the less time a participant takes to make a decision about a sensing task signifies the better timeliness and the higher probability of the participant choosing the task, and the platform tends to select this participant in order to complete the task accurately and reliably. However, participant selecting cannot be only based on the timeliness. Because the platform utility is affected by the participant utility due to the important factor of data quality, the high participant utility signifies the accurate sensing data and high benefits for the platform. Therefore, it is necessary to analyze the impact of participant utility on service ability. In terms of the task complexity and the desired rewards of participants, if the task complexity and desired rewards evaluated by participants are low, the overall reward utilization rate of the platform is high and the participant service ability is also strong. According to the above analysis, the participant service ability can be measured by the timeliness, participant utility, task complexity and desired rewards.

We know participation decision time $B_{ij}$ of $p_{i}$ to $f_{j}$ is $t_{ij}^{m}-t_{ij}^{a}$ in Section 4.1, and its maximum value is $t_{f_{j}}^{e}-t_{ij}^{a}\;\;$. If participation decision time $B_{ij}$ is short, $p_{i}$ chooses the best sensing task $f_{j}$ timely. Therefore, timeliness of participants $M_{ij}$ can be analyzed by $B_{ij}$. Obviously, when $B_{ij}$ is equal to 0, timeliness $M_{ij}$ is the maximum and $M_{ij}$ must decrease with the growing $B_{ij}$. Moreover, when the value of $B_{ij}$ is small, the task duration is relatively long and the value of $M_{ij}$ changes slightly around the maximum value. Otherwise, when the value of $B_{ij}$ is large, the sensing time allowed by the platform is limited and the value of $M_{ij}$ will drop sharply. Although the above-mentioned nonlinear trend is consistent with $1-e^{-e^{-B_{ij}}}$, for the convenience of description and comparison, it is defined that the value of $M_{ij}$ is set to 1 when $B_{ij}$ is set to 0, and the value of $M_{ij}$ is set to 0 when $B_{ij}$ is equal to $t_{f_{j}}^{e}-t_{ij}^{a}\;\;$. Due to the non-linear variation trend, $M_{ij}$ decreases slowly first and then sharply decreases and the quantification of timeliness $M_{ij}$ is shown in Equation (20).

(20) $$M_{ij}=\left\{\begin{array}{c} \;\;\;\;\;\;\;\;\;\;\;\;\;\;\;\;\;\;\;\;\;\;\;\;\;\;\;\;\;\;\;\;\;\;\;\;\;\;\;\;\;\;\;\;0\;\;\;\;\;\;\;\;\;\;\;\;\;\;\;\;\;\;\;\;\;\;\;\;\;,\;\;\;\;\;t_{ij}^{a}=t_{f_{j}}^{e}\\ \frac{T_{ij}\left(B_{ij}+t_{ij}^{a}\right)-T_{ij}\left(t_{f_{j}}^{e}\right)}{T_{ij}\left(t_{ij}^{a}\right)-T_{ij}\left(t_{f_{j}}^{e}\right)}\;\;\;\;,\;\;\;\;t_{ij}^{a}\neq t_{f_{j}}^{e} \end{array}\right.$$

where $T_{ij}\left(t\right)=1-e^{-e^{-t}}$ describes the overall trend of timeliness with participation decision time.

The platform determines whether to receive sensing data according to the participant service ability. When continuing to participate in sensing tasks, the service ability of participants is updated constantly, and the current service ability depends on not only the next sensing task but also the utility of the participants who completed the last sensing task. Although participants select the best sensing task at the initial stage, the historical task list is empty, which means that there is no participant who completed the last sensing task. Therefore, the initial service ability is evaluated according to the timeliness, task complexity and desired rewards. The range of service ability is defined in [0,1], and the value of the strongest service ability is 1. Assuming the initial best sensing task of participant $p_{i}$ is $f_{j}$, we can obtain initial service ability $s_{i}^{j}$ of $p_{i}$ by Equation (21).

(21) $$s_{i}^{j}=M_{ij}\cdot \frac{\min(c_{kj})}{c_{ij}}\cdot \frac{\max\left(D_{kj}\right)-D_{ij}}{\max\left(D_{kj}\right)-\min\left(D_{kj}\right)},\;\;\;\;\forall p_{k}\in P^{j}$$

When the historical task list is not empty, if participant $p_{i}$ completes the last sensing task significantly better than other participants, the platform will choose to receive the sensing data from $p_{i}$, which means $p_{i}$ has the strongest service ability to perform the next sensing task. With the increase of participant utility, a new equilibrium is reached between participants and platform, the more advantageous to platform completing the sensing task reliably, so the service ability of participants grows faster and faster. Therefore, the utility of $p_{i}$ completing the last sensing task $f_{b}$ is assumed to be $u_{b}^{p_{i}}$. According to the initial service ability quantification method and the value range of service ability, if $p_{i}$ selects $f_{h}$ as the next sensing task, next service ability $s_{i}^{h}$ can be obtained by Equation (22).

(22) $$s_{i}^{h}=\min\left(1,s_{i}^{h}\cdot e^{\left(u_{b}^{{}^{\prime }p_{i}}-1\right)}\right)$$

In Equation (22), $u_{b}^{{}^{\prime }p_{i}}$ is equal to ubpi∑ubpi∑ubpiPbPb, where ∑ubpi∑ubpiPbPb is the average utility of participants, so $u_{b}^{{}^{\prime }p_{i}}$ representing the extent to which utility $u_{b}^{p_{i}}$ of $p_{i}$ is significantly higher than those of other participants for last sensing task $f_{b}$.

## 5. Data Collection Mechanism

Due to the limited residual energy of mobile devices and task duration, the sensing data may not be directly transmitted to the platform, so the transmission modes of sensing data can be switched according to the relationship between the theoretical transmission delay and the maximum transmission delay. Besides, participants choose the best relay according to the next service ability, similarity of sensing tasks and intimacy degree of participants. The participant service ability aware data collection mechanism is designed for the multitasking scenario.

### 5.1. Transmission Mode Determination

In the task duration, for the platform to collect sensing data in time and provide reliable services, the sensing data are transmitted directly or indirectly to the platform based on factors such as the energy consumption rate of mobile devices [28]. Therefore, it is necessary to discriminate the transmission modes of sensing data.

Constrained by the residual energy of mobile devices and sensing task duration, if participants choose to transmit sensing data directly, the energy loss of data transmission must be lower than the residual energy and the sensing data should be successfully transmitted to the platform. Therefore, under the above two conditions, the low energy consumption of transmitting the sensing data signifies the small actual transmission delay and the great probability of transmitting the sensing data directly. Otherwise, the sensing data will be transmitted indirectly through the best relay.

To discriminate the transmission modes of sensing data, we assume that all participants theoretically analyze the direct transmission. Once the above conditions are not met, participants choose the indirect data forwarding. For sensing task $f_{j}$, participant $p_{i}$ is assumed to start transmitting sensing data at time $t_{ij}^{g}$. If the sensing data transmission can be completed theoretically at time $t_{ij}^{o}$, the total volume of sensing data collected successfully is $S_{ij}$. In addition, the average data transmission rate of $p_{i}$ is assumed to be $\eta_{ij}$, so the above conditions are shown in Equation (23).

(23) $$\left\{\begin{array}{c} \gamma_{ij}\left(t_{ij}^{o}-t_{ij}^{g}\right)\leq E_{i}^{r}\\ \eta_{ij}\left(t_{ij}^{o}-t_{ij}^{g}\right)\geq S_{ij}\\ 0<t_{ij}^{o}-t_{ij}^{g}\leq t_{f_{j}}^{e}-t_{ij}^{g} \end{array}\right.$$

In Equation (23), $t_{f_{j}}^{e}-t_{ij}^{g}$ represents the maximum transmission delay allowed by the platform to collect the sensing data from $p_{i}$ and $t_{ij}^{o}-t_{ij}^{g}$ is the theoretical transmission delay. When the transmission delay is too large to meet the conditions in Equation (23), indirect forwarding is employed to complete the sensing data transmission. If the theoretical transmission delay satisfies the above equation, the sensing data can be directly transmitted to the platform. However, since the actual scenario is complicated, when the actual transmission delay is larger, participants consume more energy, which affects the timeliness and efficiency of sensing data collection. According to Equation (23), the sensing data are transmitted directly when (tijo−tijg)(tijo−tijg)(tfje−tijg)(tfje−tijg)∈(0,0.5]. Otherwise, the sensing data are transmitted in an indirect forwarding manner.

After discriminating the transmission modes of sensing data, $P_{d}=\left\{p_{d}^{1},\;\;\;\;p_{d}^{2},\;\;\;\;\cdots \right\}$ and $P_{nd}=\left\{p_{nd}^{1},\;\;\;\;p_{nd}^{2},\;\;\;\;\cdots \right\}$ are assumed to be the participants with direct transmission and indirect transmission respectively. Participant $p_{d}^{\alpha }$ transmits sensing data to the platform directly, whereas participant $p_{nd}^{\beta }$ selects the best relay from $P_{d}=\left\{p_{d}^{1},\;\;\;\;p_{d}^{2},\;\;\;\;\cdots \right\}$ to complete the sensing data transmission. If the next service ability of $p_{d}^{\alpha }$ is strong, the probability that the utilities of the participant and platform are maximized is high, and the platform will be highly likely to receive sensing data from $p_{d}^{\alpha }$, and therefore the probability of $p_{nd}^{\beta }$ choosing $p_{d}^{\alpha }$ for data transmission is high. However, in the multitasking scenario, many sensing tasks that can be selected by participants at the same time, when the best tasks selected by $p_{d}^{\alpha }$ and $p_{nd}^{\beta }$ are different, because the next service ability of $p_{d}^{\alpha }$ is stronger, $p_{d}^{\alpha }$ can successfully transmit the sensing data of $p_{nd}^{\beta }$ to the platform, but the platform cannot identify that the sensing data of $p_{d}^{\alpha }$ and $p_{nd}^{\beta }$ belong to different sensing task, the accuracy of sensing results is affected. Therefore, $p_{nd}^{\beta }$ tends to select the participant in the same sensing task for data transmission. In other words, the high similarity of sensing tasks selected by $p_{d}^{\alpha }$ and $p_{nd}^{\beta }$ signifies the great probability of $p_{nd}^{\beta }$ selecting $p_{d}^{\alpha }$ for indirect data forwarding. In addition, when $p_{d}^{\alpha }$ and $p_{nd}^{\beta }$ choose the same sensing task,$p_{d}^{\alpha }$ is not necessarily willing to help $p_{nd}^{\beta }$ forward the sensing data, because they may distrust each other and their mobile devices have the energy consumption in the process of data transmission. Therefore, $p_{d}^{\alpha }$ is not necessarily the best relay for $p_{nd}^{\beta }$. Due to the social attributes of participants, when $p_{nd}^{\beta }$ chooses the best relay or $p_{d}^{\alpha }$ helps the data forwarding, they have certain preferences [29,30,31]. If the social relationship between $p_{d}^{\alpha }$ and $p_{nd}^{\beta }$ is close, they will trust each other and $p_{nd}^{\beta }$ will be highly likely to select $p_{d}^{\alpha }$ as the best relay. Eventually, the best relay can be selected according to the next service ability of participants, similarity of sensing tasks and intimacy degree between participants.

According to the evaluation method in Equation (22), the next service ability of $p_{d}^{\alpha }$ is assumed to be $s_{d\_\alpha }^{h}$. In terms of sensing tasks similarity, participants only consider the task complexity and desired reward from their own perspectives when selecting the best sensing task, and they ignore the choices of other participants. In other words, participants choose the best sensing task without distinguishing the transmission mode. Therefore, they cannot analyze the similarity between sensing tasks of all other participants within the participation decision time. To avoid the above problem, participants perform sensing tasks continuously in the sensing area. When the similarity between historical task lists of participants is high, they are interested in many sensing tasks and have high similarity in the next sensing task. When analyzing the best relay, we should measure the similarity of sensing tasks according to historical task lists. The method measuring similarity $r_{\alpha \beta }$ between sensing tasks of $p_{d}^{\alpha }$ and $p_{nd}^{\beta }$ is shown in Equation (24).

(24) $$r_{\alpha \beta }=\frac{\left|F^{\alpha }\cap F^{\beta }\right|}{\left|F^{\alpha }\cup F^{\beta }\right|}$$

where $F^{\alpha }$ and $F^{\beta }$ represent the historical sensing tasks performed by $p_{d}^{\alpha }$ and $p_{nd}^{\beta }$, respectively, $\left|F^{\alpha }\cap F^{\beta }\right|$ and $\left|F^{\alpha }\cup F^{\beta }\right|$ represent the total numbers of $p_{d}^{\alpha }$ and $p_{nd}^{\beta }$ performing the same historical sensing tasks, and $r_{\alpha \beta }$ has value range [0,1].

In addition, the intimacy degree between participants can be analyzed through the interaction time, because the long interaction time between participants signifies the high intimacy degree between them. The intimacy degree between participants grows fast when their early interactions are established. As they continue to interact, the intimacy degree gradually increases to the maximum. When the intimacy degree is stable, its value remains almost unchanging. Therefore, the range of intimacy degree is defined in [0,1], and the intimacy degree between $p_{d}^{\alpha }$ and $p_{nd}^{\beta }$ can be obtained according to the interaction time, as shown in Equation (25).

(25) Nαβ=nαβnαβ∑pdσ∈Pdnσβ∑pdσ∈Pdnσβ1+e−t

where $n_{\alpha \beta }$ represents the times that $p_{d}^{\alpha }$ helps $p_{nd}^{\beta }$ complete the sensing data transmission, and $\sum_{p_{d}^{\sigma }\in P_{d}}n_{\sigma \beta }$ represents the times of the participants in set $P_{d}$ forwarding the sensing data for $p_{nd}^{\beta }$. To accurately determine the initial intimacy degree between participants, when $p_{d}^{\alpha }$ and $p_{nd}^{\beta }$ establish the first interaction, their intimacy degree is set to nαβnαβ∑pdσ∈Pdnσβ∑pdσ∈Pdnσβ.

Since the next service ability of $p_{d}^{\alpha }$ is $s_{d\_\alpha }^{h}$, similarity of sensing tasks $r_{\alpha \beta }$ and intimacy degree $N_{\alpha \beta }$ between $p_{d}^{\alpha }$ and $p_{nd}^{\beta }$ are described by Equations (24) and (25), respectively. Finally, $p_{nd}^{\beta }$ chooses the best relay according to Equation (26).

(26) $$p_{d}^{\alpha }=\underset{\forall p_{d}^{\alpha }\in P_{d}}{\arg}\max\;\;\left(s_{d\_\alpha }^{h}\cdot r_{\alpha \beta }\cdot N_{\alpha \beta }\right)$$

The platform determines whether to receive the sensing data of participants according to the service ability. If the best relay $p_{d}^{\alpha }$ is selected by $p_{nd}^{\beta }$ according to Equation (26), $p_{d}^{\alpha }$ not only transmits its own sensing data, but also helps $p_{nd}^{\beta }$ forward sensing data. Therefore, after receiving the sensing data from $p_{nd}^{\beta }$, $p_{d}^{\alpha }$ marks it for the platform to collect the sensing data correctly.

### 5.2. Sensing Data Collection

In the multitasking scenario, participants select the best sensing task according to the sensing task complexity and desired rewards to maximize their utilities with the minimum energy consumption. However, the platform determines whether to receive the sensing data to maximize its utility and complete sensing tasks efficiently and accurately by evaluating the service ability of participants. The next sensing task that participant $p_{i}$ selects to perform is assumed to be $f_{h}$ and the platform selects participants according to Equation (27).

(27) $$\begin{array}{c} \left\{\begin{array}{c} \max\;\;\;\;u_{h}^{s}\left(q_{ih},\;\;\;m_{p}\right)\\ \max\;\;\;\;u_{h}^{p_{i}}\left(q_{ih},\;\;\;m_{p}\right)\\ \max\;\;\;\;(\min\;\;\;\;s_{i}^{h}) \end{array}\right.\\ s.t.\;\left\{\begin{array}{c} x_{ih}=1\\ \sum_{\forall p_{i}\in P^{h}}c_{ih}^{g}\leq R_{h},\;\;\;\;\forall p_{i}\in P^{h}\\ B_{ih}<t_{f_{h}}^{e}-t_{ih}^{a} \end{array}\right. \end{array}$$

In Equation (27), objective function $\max\;\;\;\;u_{h}^{s}\left(q_{ih},\;\;\;m_{p}\right)$ and $\max\;\;\;\;u_{h}^{p_{i}}\left(q_{ih},\;\;\;m_{p}\right)$ are employed to maximize the utilities of participants and platform, when participants select the best sensing task and platform chooses the participants with strong service ability. Besides, $\max\;\;\;\;(\min\;\;\;\;s_{i}^{h})$ represents the requirements of the platform for participant service ability. The constraint conditions mean that, when participant $p_{i}$ chooses $f_{h}$ as the next sensing task, the participation decision time of $p_{i}$ must meet the real time requirement of $f_{h}$ and cannot exceed the maximum participation decision time of $f_{h}$. Furthermore, the total rewards of all participants who choosing $f_{h}$ must not exceed the total reward cost of $f_{h}$. Participants and platform choose each other to transmit sensing data directly or indirectly, and then the platform chooses to receive the sensing data from participants with strong service ability. The algorithm of proposed PSAA data collecting mechanism is shown below (Algorithm 1).

**Algorithm 1** PSAA data collecting mechanism.
1:BEGIN2:
**for all** $p_{i}\in P$ **do**
3: choose task from *F* of $p_{i}$ according to Equation (2)4:
**end for**
5:
**for all** $p_{\alpha }^{j}\in P^{j}$ **do**
6: determine sending method of $p_{\alpha }^{j}$ according to Equation (23)7:
**end for**
8:
**for all** $p_{nd}^{\gamma }\in P_{nd}$ **do**
9: **for all** $p_{d}^{\lambda }\in P_{d}$ **do**10:  **if** satisfy Equation (26) **then**11:   $p_{nd}^{\gamma }$ sends data to $p_{d}^{\lambda }$12:  **else**13:   Go to Line 914:  **end if**15: **end for**16:
**end for**
17:
**for all** $p_{\alpha }^{j}\in P^{j}$ **do**
18: **if** satisfy Equation (27) **then**19:  the server accepts sensing data20: **else**21:  the server refuses to receive22: **end if**23:
**end for**
24:END


## 6. Numerical Results

We evaluated the proposed mechanism on a real dataset: T-drive [32,33]. T-drive contains the GPS traces of 10,357 taxis in Beijing for one week, from 2 February to 8 February 2008. Each taxi is equipped with the GPS sensor, and the average retrieving the GPS position is about 177 s. In T-drive, each trace includes the taxi ID, timestamp and the latitude and longitude position of the taxi. We took each taxi as a participant equipped with built-in sensors and assumed that the data collected by the participants are their GPS location information.

We chose the region around the Fourth Ring Road in Beijing as the sensing region, the latitude range of the sensing region is (39.84002${}^{\circ }$ N–39.99397${}^{\circ }$ N) and the longitude range is (116.27621${}^{\circ }$ E–116.49424${}^{\circ }$ E). According to the location of the sensing region, we selected 900 participants whose trajectories were distributed in the sensing region. In addition, for the convenience of analysis, a dataset containing the GPS traces of 900 participants for one day was extracted from T-drive. Meanwhile, a false dataset containing 100 participants uploading the wrong GPS location information was constructed to simulate the dishonesty behaviors of participants collecting low-quality sensing data. We selected another 100 participants in the sensing region from T-drive. Some of their GPS position were modified during a certain period, and the modified GPS position was randomly generated. Therefore, we used the real dataset and the false dataset to build the simulation environment. Considering that a participant’s reward could be achieved in different formats in practice, such as money or bonus points, we used dimensionless units to represent both the participants’ reward and the maximum total reward of sensing task. The main simulation parameters are given in Table 1.

As shown in Table 1, the residual energy of participants and the reward of a single participant were assumed to be generated randomly, and all simulations were run on MATLAB, which was deployed on a PC with 1.70 GHz CPU and 4GB RAM. In addition, the proposed PSAA was compared with the ProMoT [17] and IIM [20] mechanisms. First, we randomly selected some participants to observe the change of their service ability. Then, the utilities of participants and platform were analyzed to validate PSAA. Furthermore, in the multitasking scenario, PSAA, ProMoT and IIM mechanisms were analyzed under different numbers of participants and different overall reward expenses.

### 6.1. Utility Analysis of Participants and Platform

**Figure 2:**
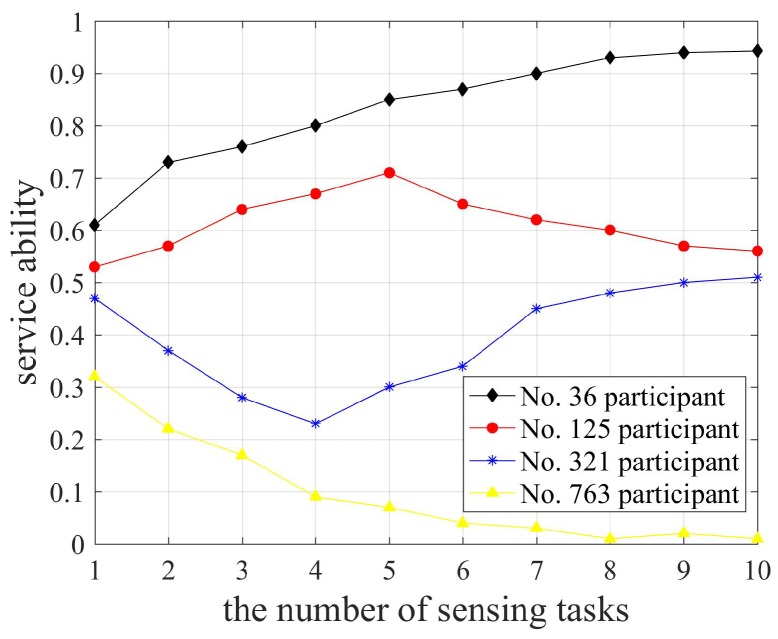
Changes in the service ability of participants.

To effectively analyze the service ability of participants, we selected participants No. 36, No. 125, No. 321 and No. 763 who performed the same sensing tasks, and their service abilities were clearly differentiated. The changes in the service ability are shown in Figure 2. Obviously, the range of service ability is [0,1] according to its definition. We note a difference in the initial service ability of participants, which may be due to factors such as the residual energy or desired reward. Besides, the service ability of participant No. 36 increased gradually from 0.61 to 0.93, indicating that he chose the best sensing task and collected high-quality sensing data, so his service ability increased. The service ability of participant No. 763 reduced gradually from 0.32 to 0.01, which may be caused by dishonest behavior and unreasonable desired reward. The service ability of participant No. 125 increased from 0.53 to 0.7 and then decreased to 0.55. It is possible that the residual energy of participant No. 125 was insufficient to perform more sensing tasks, resulting in the increased complexity of the sensing task. The service ability of participant No. 321 reduced from 0.47 to 0.23 and then increased to 0.51, because it may be difficult to select the best sensing task when the number of sensing tasks is small, resulting in his low willingness. As the number of tasks increases, he can choose the best sensing task to increase the service ability.

**Figure 3:**
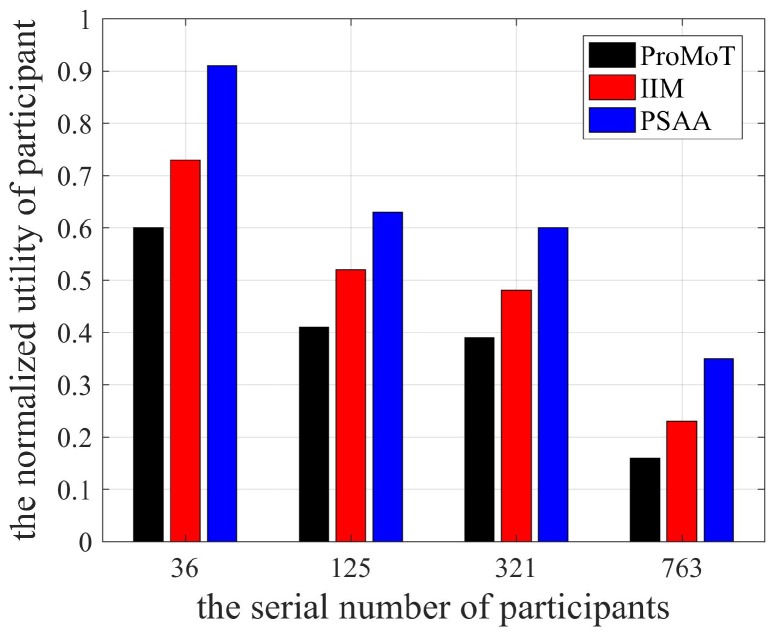
The utility of participants.

The utility of participants is shown in Figure 3. It can be seen that the utility of participants in PSAA was the highest because participants chose the best task and platform selectively collects sensing data. In the bilateral selection process, the Stackelberg game was formed with an equilibrium solution to provide participants with the highest utility. At the same time, compared to ProMoT, IIM had higher utility of participants, because the platform selectively received the sensing data according to the reverse auction principle and participants collected high quality sensing data to obtain as high a reward as possible.

**Figure 4:**
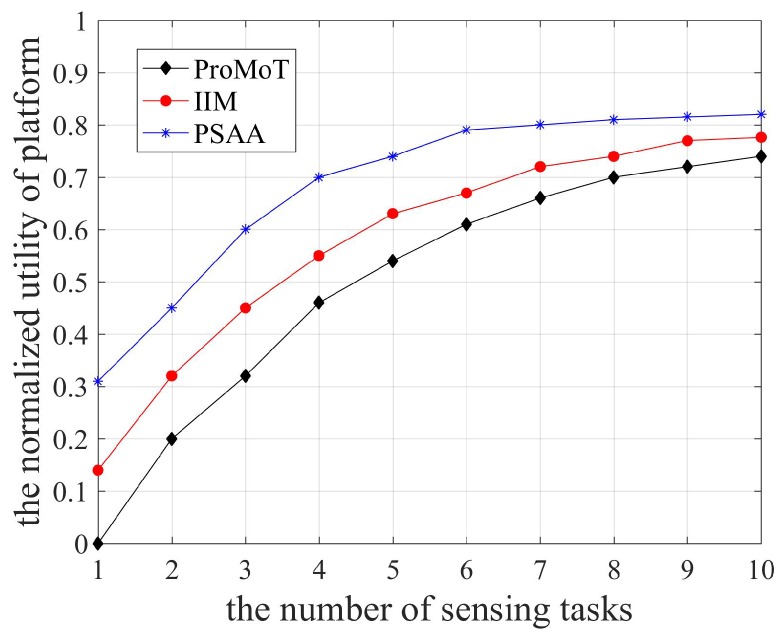
The utility of platform.

The platform utility is shown in Figure 4. As the number of sensing tasks increased, the platform utility increased gradually and then remained relatively stable, because the platform needed to collect, analyze, and process the sensing data. In addition, PSAA stabilized before IIM and ProMoT. Because PSAA takes into account the timeliness of participants and data transmission delay, it consumes the least time to complete the sensing task and its platform utility also stabilizes quickly. Since the platform selectively receives sensing data according to the participant service ability, the utilities of platform and participants of PSAA are both the highest due to the equilibrium solution. Compared to IIM, the platform in ProMoT only considers the total volume of sensing data, which has the lowest utility.

### 6.2. Performance Analysis in the Multitasking Scenario

We assumed the total number of sensing tasks is 5 in the multitasking scenario, as shown in Table 2. PSAA, ProMoT and IIM are verified based on the total volume of sensing data, overall sensing time, total number of participants in each task and overall reward expenses.

#### 6.2.1. Performance Analysis under Different Numbers of Participants

**Figure 5:**
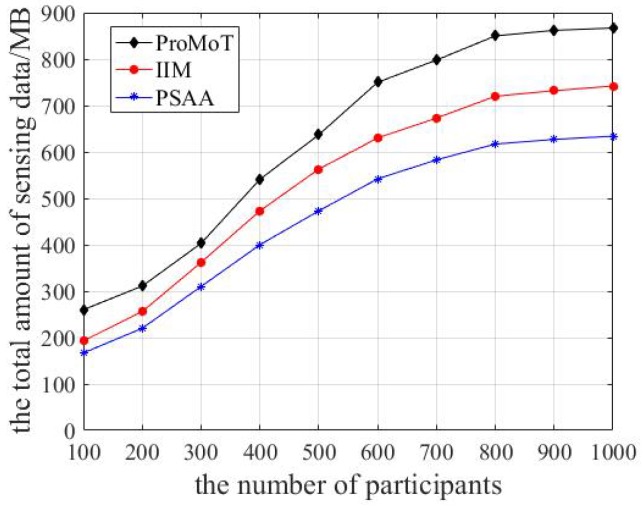
The total volume of sensing data.

The total volume of sensing data collected by the three data collection mechanisms is shown in Figure 5. It can be seen that the total volume of sensing data increased rapidly and then slowly and finally stabilized with the growing number of participants, because the number participants was small at the beginning and the platform actively received sensing data to complete the sensing task efficiently and accurately. When the number of participants increased gradually, there was some redundancy between sensing data and the platform only processed the valid sensing data, so the total volume of sensing data grew slowly. When the number of participants is large, the overall reward expense of the platform was limited and the sensing data quality among participants was different, which means that the platform needed to reject some participants’ sensing data, and therefore the total volume of sensing data remained stable. In addition, ProMoT only considers the total volume of sensing data collected by the platform, so its total volume of sensing data is the largest. However, compared to IIM, PSAA analyzes the data transmission delay based on the total volume of sensing data, so its total volume of sensing data is the least.

**Figure 6:**
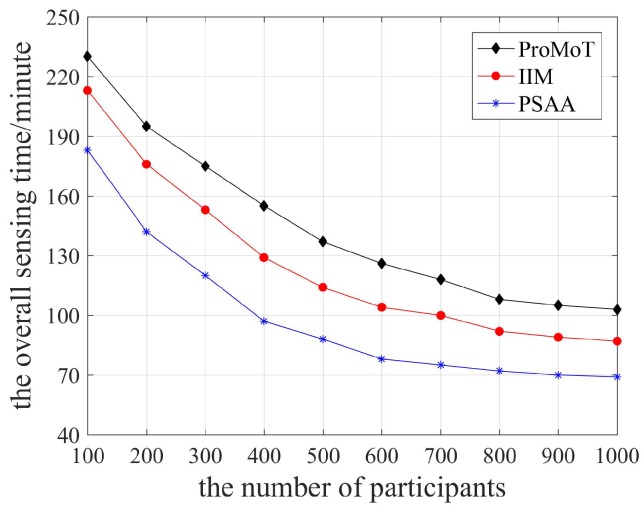
The overall sensing time.

The overall sensing time of the three data collection mechanisms is shown in Figure 6. Apparently, the overall sensing time decreased rapidly and then stabilized with the growing number of participants. When the number of participants started to increase, the total volume of sensing data collected by the platform also grew to facilitate the completion of sensing tasks and reduce the overall sensing time. When the number of participants was large enough, the total volume of sensing data processed by the platform did not increase, so the overall sensing time stabilized. In addition, the timeliness of participants is considered by PSAA in the service ability analysis and the transmission mode of sensing data is determined according to the transmission delay, so the overall sensing time of PSAA is the lowest. IIM updates the participant reputation dynamically and selects participants with high reputation, which has lower overall sensing time than ProMoT.

**Figure 7:**
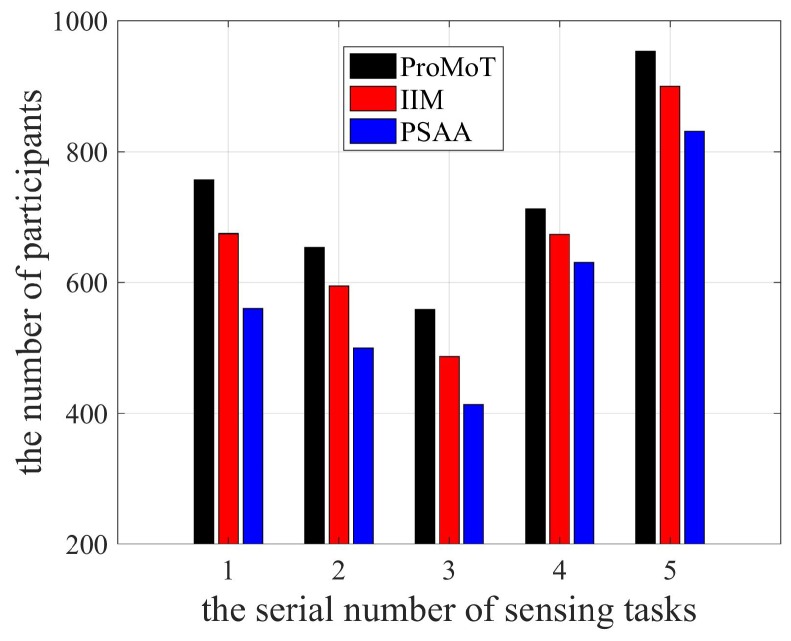
The number of participants.

The number of participants in each sensing task is shown in Figure 7. It can be seen that the number of participants in sensing task No. 5 was the largest for all three mechanisms, because the platform allowed the longest sensing time for task No. 5 due to the large and complex scene scale. Therefore, the platform required many participants in No. 5 scene. Moreover, participants select the best sensing task and the platform chooses sensing data according to the participant service ability in PSAA, so its number of participants is the smallest. The reputation of participants is analyzed by the platform in IIM, whereas ProMoT only maximizes the rewards of participants, resulting in the larger number of participants in ProMoT.

#### 6.2.2. Performance Analysis under Different Total Reward Expenses

**Figure 8:**
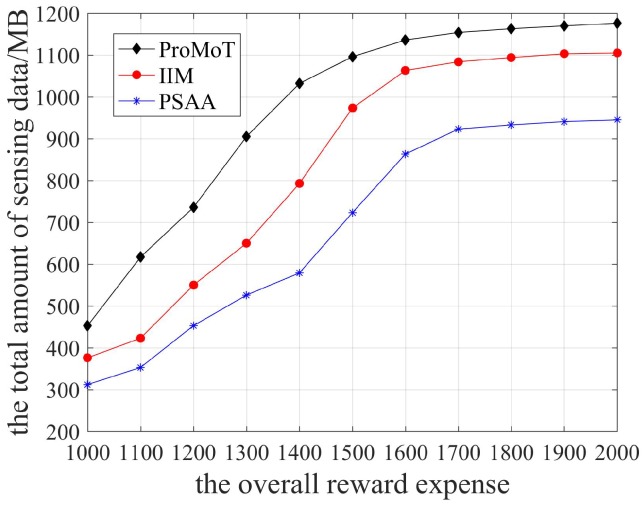
The total volume of sensing data.

The total volume of sensing data collected by the three data collection mechanisms is shown in Figure 8. It can be seen that the total volume of sensing data grew to a certain extent and then stabilizes with the increase of overall reward expense. When the overall reward expense increased gradually, it inevitably attracted participants to perform the sensing task actively, so the total volume of sensing data grew gradually. When the overall reward expense was high, the platform selectively received the sensing data under the three data collection mechanisms, so the total volume of sensing data stabilized. In addition, the platform expects to collect enough sensing data in ProMoT, so its total volume of sensing data is the largest. Compared to IIM, PSAA considers the data transmission delay, so its total volume of sensing data collected by the platform is the least.

**Figure 9:**
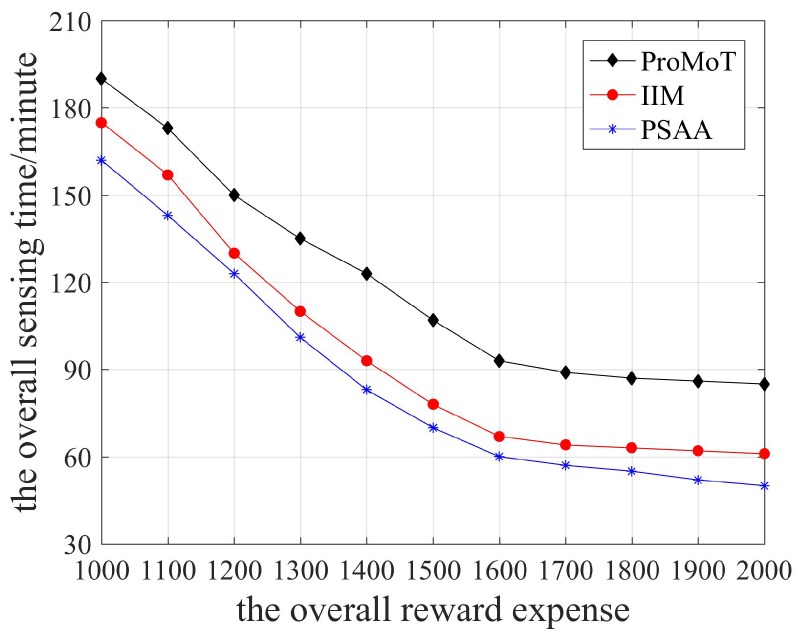
The overall sensing time.

The overall sensing time of the three data collection mechanisms is shown in Figure 9. It can be seen that the overall sensing time decreased and then stabilized with the increase of overall reward expense. When the overall reward expense was low and the number of participants was small, the platform could not collect sensing data efficiently, resulting in a high overall sensing time. However, more and more participants chose to perform sensing tasks due to the increase of overall reward expense, so the overall sensing time decreased. When the overall reward expense was relatively large, the total volume of sensing data stabilized, which means the platform collected no more sensing data, so the overall sensing time remained almost unchanged. In addition, the utility of participants and platform in PSAA can reach the maximum and its overall sensing time stabilizes first. While aiming to maximize the utility of participants, ProMoT has the largest overall reward expense.

**Figure 10:**
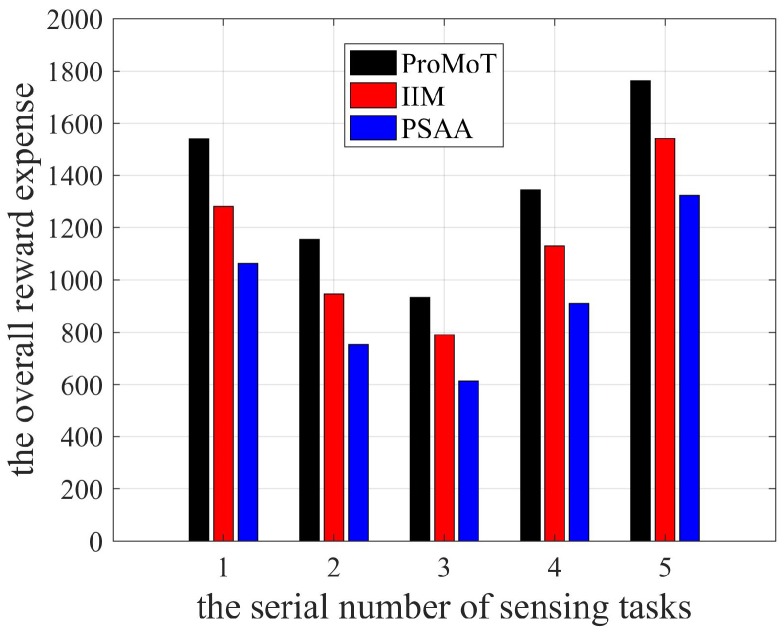
The overall reward expense.

The overall reward expense of each sensing task is shown in Figure 10. Obviously, the overall reward expense of sensing task No. 5 was the largest for all three mechanisms. According to Figure 6, the number of participants in sensing task No. 5 was the largest, so it had the largest overall reward expense. Combined with the number of participants in Figure 6, Figure 9 shows the overall reward expenses of PSAA and ProMoT are the lowest and the highest, respectively.

## 7. Conclusions

To collect data efficiently and reliably in MCS, PSAA is proposed in our paper. First, participants select the best sensing task by measuring the task complexity and desired reward. Second, the Stackelberg game model is established according to the mutual choice between participants and the platform to maximize the utilities of participants and platform. Then, the next service ability of participants is employed along with the initial service ability for the platform to selectively receive sensing data. Finally, participants transmit data directly or indirectly to the platform, ensuring the efficient and accurate completion of sensing tasks the minimum overall reward expense. In our future work, the mobility of participants would be considered for MCS data collection.

## Figures and Tables

**Table 1:** Simulation parameter settings.

Parameter	Value
simulation duration/s	34,200
number of participants	[100, 1000]
number of tasks	[1, 15]
task duration/s	[900, 3600]
total energy of mobile device/(mA·h)	2000
residual energy of mobile device/(mA·h)	[400, 1900]
maximum total reward of sensing task	[1000, 2000]
reward for a single participant	[1, 5]
average data transmission rate/kbps	400

**Table 2:** Sensing task information.

Task	Starting Time	Ending Time	Task Duration/min
1	08:00:00	08:50:00	50
2	08:07:00	08:47:00	40
3	08:12:00	08:42:00	30
4	08:26:00	09:11:00	45
5	08:40:00	09:40:00	60
